# Global Population Exposure to Extreme Temperatures and Disease Burden

**DOI:** 10.3390/ijerph192013288

**Published:** 2022-10-14

**Authors:** Yajie Du, Ming Jing, Chunyu Lu, Jingru Zong, Lingli Wang, Qing Wang

**Affiliations:** 1 Department of Biostatistics, School of Public Health, Cheeloo College of Medicine, Shandong University, Jinan 250012, China; 2 National Institute of Health Data Science of China, Shandong University, Jinan 250012, China; 3 School of Computer Science and Technology, Qilu University of Technology (Shandong Academy of Science), Jinan 250353, China

**Keywords:** population exposure, extreme temperature, disease burden, low-income and middle-income countries

## Abstract

The frequency and duration of extreme temperature events continues to increase worldwide. However, the scale of population exposure and its quantitative relationship with health risks remains unknown on a global scale, limiting our ability to identify policy priorities in response to climate change. Based on data from 171 countries between 2010 and 2019, this study estimated the exposure of vulnerable populations to extreme temperatures, and their contemporary and lag associations with disease burden attributed to non-optimal temperatures. Fixed-effects models and dynamic panel models were applied. Increased vulnerable population exposure to extreme temperatures had adverse contemporary effects on the burden of disease attributed to non-optimal temperature. Health risks stemming from extreme cold could accumulate to a greater extent, exhibiting a larger lag effect. Population exposure to extreme cold was mainly distributed in high-income countries, while extreme heat occurred more in low-income and middle-income countries. However, the association between population exposure to extreme cold and burden of disease was much stronger in low-income and middle-income countries than in high-income countries, whereas the effect size of population exposure to extreme heat was similar. Our study highlighted that differential strategies should be determined and implemented according to the characteristics in different countries.

## 1. Introduction

The frequency, intensity, and duration of extreme temperature events are expected to rise worldwide, prompting a sharp increase in population exposure to extreme temperatures in the coming decades [1,2,3,4]. Although several studies have documented the health risks associated with extremely cold and extremely hot temperatures, a globally comprehensive understanding of population exposure to extreme temperatures is lacking [5,6,7,8,9], and the scale of population exposure and its quantitative relationship with health risks remain unknown. This restricts our ability to identify how to distribute resources to deal with emerging health risks (reducing population-wide exposure versus increasing the resistance of vulnerable populations) [1,3,10].

Numerous epidemiological studies have demonstrated that both extreme heat and cold have an adverse influence on a wide range of health outcomes in different regions of the world [5,6,9,11,12,13]. Health indicators include all-cause and cause-specific mortality, morbidity to emergency department visits, hospitalizations, and burden of disease for specific diseases (such as cardiovascular and respiratory diseases) [12,14,15,16,17]. Measurements of extreme heat and cold started using a single meteorological index, and then a composite temperature index was developed to capture the apparent temperature based on thermal equilibrium [2,14,18,19]. Furthermore, the effects of extreme temperature have been found to be heterogeneous [1,9,11,20]. Despite there being several studies in low-income and middle-income countries (LMICs), an increasing number of studies are reporting that low socioeconomic status (SES) is associated with stronger temperature-related mortality, because LMICs lack the human and financial resources to deal with extreme temperatures [7,21].

However, a comprehensive understanding of the association between population exposure to extreme temperatures and health risks is lacking. Relative risk measurements (such as relative risk, odds ratio, and percentage change) are conventionally used to assess the dose–response association between temperature indicators and health outcomes. Nevertheless, relative risk fails to capture population exposure [14]. A high relative risk cannot identify the extent of temperature-associated outcomes resulting from the low prevalence of exposure or vulnerability of exposed populations within a given region [14]. Recent studies have adopted an attributable fraction or number to integrate relative risk exposure with the prevalence of exposure [5,20,21]. Doing so makes it possible to capture the extent to which health outcomes can be attributed to non-optimum temperatures; however, this leaves open the question of the relationship between population exposure to extreme temperature and associated health loss.

To address this issue, based on data from 171 countries during 2010–2019, we assessed the temporal trend in global population exposure to extreme temperatures and its association with disease burden. It is often assumed that susceptibility to extreme temperatures varies according to the SES. Thus, we conducted a sub-sample analysis in high-income countries (HICs) and LMICs. This study has important implications for understanding the potential impact of population exposure to extreme heat and cold.

## 2. Methods

### 2.1. Burden of Disease Attributed to Non-Optimal Temperature

The disease burden attributed to non-optimal temperature in our study was measured by cause-specific disease mortality and years of life lost (YLL) attributable to high or low temperature. Cause-specific diseases include all-cause, cardiovascular diseases (CVD), and chronic respiratory diseases (CRD). Mortality and YLL were also divided by age group (e.g., age-standardized rate, children (under 5), and the elderly (age 55+)). The burden of disease attributed to non-optimal temperature data was downloaded from the Global Burden of Disease Study (GBD 2019) Results Tool database (http://ghdx.healthdata.org/gbd-results-tool, accessed on 8 March 2022).

### 2.2. Population Exposure to Extreme Heat and Cold

This study used the vulnerable population’s annual exposure to extreme heat and cold as the main exposure variables. Vulnerable population refers to the elderly (55+) and children (<5). The spatial distribution of the vulnerable population was calculated by combining data from the Applications Center Gridded Population of the World Version 4 with the United Nations World Population Prospects [3]. The exposure-weighted mean was applied to measure population exposure to extreme temperature, that is, the number of days of extreme temperature multiplied by the number of vulnerable people affected, divided by the total number of vulnerable populations in the country. This could increase comparability by excluding differences in population growth and demographic changes between countries. In this study, the universal thermal climate index (UTCI) was used as the temperature exposure metric, and a daily maximum UTCI greater than 32 °C was defined as extreme heat and a daily minimum UTCI of less than −13 °C as extreme cold. The UTCI refers to the air temperature of a reference environment that would cause the same physiological response (sweat production; shivering; skin wittedness; skin blood flow; and rectal, mean skin, and face temperatures) in the human body as the actual environment [22]. The UTCI has been recognized as a universal tool for investigating the adverse effects of heat and cold exposure on human health over a wide area with different environmental characteristics [23]. UTCI data were collected from ERA5-HEAT [24]. Details of the calculation of disease burden and exposure-weighted mean are presented in the Supplementary Materials (Texts S1 and S2).

### 2.3. Statistical Analysis

One hundred and seventy-one countries for which the above data were available were classified into an HICs group and an LMICs group according to the World Bank classification in 2017 [25]. Incomplete data were imputed using the multiple imputation method using chained equations (Text S4). Continuous variables were reported as median (interquartile range, IQR). In addition, the non-parametric Mann–Whitney U test was applied to compare indicators between the HICs group and the LMICs group.

A two-way fixed effects model was used to examine the association between vulnerable population exposure to extreme temperature and disease burden attributed to non-optimal temperature. The model has the following form:

(1) $$Y_{i,t}=\alpha +\beta X_{i,t}+\gamma X_{i,t}^{\prime }+\nu_{i}+\mu_{t}+\varepsilon_{i,t}$$

where $Y_{i,t}$ is the annual age-adjusted mortality or YLL attributed to extreme heat and cold in country *i* at time *t*, $X_{i,t}$ is the exposure weighted mean of the vulnerable population to extreme temperature in country *i* at time *t*, β refers to the contemporary effects of extreme temperature, and $X_{i,t}^{\prime }$ is a set of control variables, including urbanization, gross domestic product per capita, the International Health Regulations (IHR) core capacities score, and particulate matter exposure. The IHR core capacities score was proposed by the World Health Organization to reflect a country’s potential capacity with respect to detection, preparedness and response to public health risks and emergencies of national and international concern. Details for covariates were presented in Supplementary Materials (Text S3). The model includes both location-specific fixed effects $\nu_{i}$ and time-specific fixed effects $\mu_{t}$. In addition, $\varepsilon_{i,t}$ is the residual term.

In addition, the study used Fisher’s permutation test to identify the significance of coefficient differences between the two income groups. The empirical *p*-values were calculated by bootstrap testing. They were estimated grounded on the null hypothesis that the coefficients between the two groups would be equal. In addition, the alternative hypothesis was that the first group coefficient would be greater than the second one.

Subsequently, a dynamic panel model was built to estimate the cumulative effects of extreme temperature exposure on mortality and YLL. Before estimating the model, the Levin–Lin–Chu and Im–Pesaran–Shin tests rejected the hypothesis of unit roots, showing that the transformed series were stationary. Our model was estimated using the Generalized Method of Moments (GMM). The GMM model can be shown as follows:

(2) $$Y_{i,t}=\alpha +\beta_{1}Y_{i,t-1}+\beta_{2}X_{i,t}+\gamma X_{i,t}^{\prime }+\mu_{i}+\varepsilon_{i,t}$$

where $Y_{i,t-1}$ accounts for the time series correlation and represents the age-adjusted mortality or YLL in country *i* at time *t−1*, and $\mu_{i}$ represents the country dummy variables, which account for cross-country variations. In the dynamic model, the contemporary effects of extreme temperature were calculated using $\beta_{1}$ and $\beta_{2}$.

When 0 < $\beta_{1}$ < 1, then the cumulative effects were calculated as follows [19]:

(3) $$\underset{n\to \infty }{\lim}\beta_{2}+\beta_{1}\beta_{2}+\beta_{1}^{2}\beta_{2}+\cdots =\underset{n\to \infty }{\lim}\frac{\beta_{2}\left(1-\beta_{1}^{n}\right)}{1-\beta_{1}}=\frac{\beta_{2}}{1-\beta_{1}}$$

The 95% confidence intervals (CI) of the cumulative effects were calculated through the 95% CI of the natural logarithm of the cumulative effects.

The data were analyzed using RStudio version 4.1.2 and Stata version 14.

## 3. Results

### 3.1. Global Population Exposure to Extreme Temperature and Health Burden Attributed to Non-Optimal Temperature

Legend: Diagonal stripe indicates no data (not included). The older population refers to the population aged over 55. YLL, years of life lost.

Descriptive statistics are summarized in Table 1, and a more detailed version is provided in Table S1. Measured by the exposure-weighted mean, the median values (IQR) of the older population (55+) and children (<5 years) exposed to extreme heat were 128.30 (IQR:42.98, 248.30) person-days/year^−1^ and 124 (IQR:42.06, 241.10) person-days/year^−1^, respectively. Regarding extreme cold, the median values (IQR) for exposure experienced by the older population and children were 0.00 (IQR:0.00, 19.79) and 0.00 (IQR:0.00, 18.44) person-days/year^−1^, and was relatively low in most countries. During the study period, the median (IQR) age-standardized death rates attributable to high and low temperature per 10 million people were 212.20 (IQR:19.17, 567.30) and 1443 (IQR:381.30, 2505), and the corresponding YLLs were 61.64 (IQR:4.54, 175.90) and 190.70 (IQR:46, 366.60), respectively. The attributed disease burden mainly stemmed from CVD, which contributed to approximately 14% and 66% of the disease burden at high and low temperatures, respectively.

**Figure 1:**
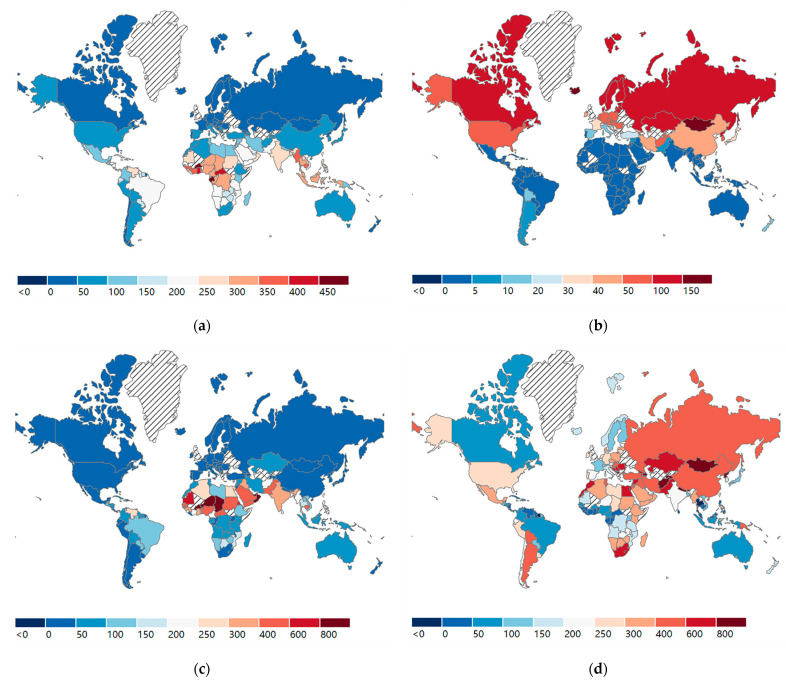
The ten-year average (2010–2019) of older population exposure to extreme temperature and associated disease burden. (**a**) Older population exposure to extreme heat (person-days/year^−1^). (**b**) Older population exposure to extreme cold (person-days/year^−1^). (**c**) YLL attributable to high temperature (per 100,000 population). (**d**) YLL attributable to low temperature (per 100,000 population).

Compared to HICs, LMICs had a much higher median value (IQR) of population exposure to extreme heat, but a level of lower population exposure to extreme cold. The disease burden attributed to non-optimal temperature exhibited a pattern similar to that of the distribution of population exposure by income group. However, the median value of disease burden attributed to high temperature was 20-fold higher in LMICs than in HICs, which is far beyond the gap in population exposure between LMICs and HICs. Conversely, although the median value of health burden attributed to low temperature was higher in HICs, the differences in the medium value of disease burden between the two countries were less than two times lower than the disparities in population exposure. The global distribution of older populations exposed to high and low temperatures and their associated YLL are presented in Figure 1. Additional information is available on the website (https://panel.nihds.com/, accessed on 26 May 2022). The distribution pattern of population exposure and health burden by income group is consistent with that suggested in Table 1. HICs exhibit a higher level of population exposure to extreme cold and corresponding health losses. In contrast, higher population exposure to extreme heat and health loss occurs in LMICs.

### 3.2. Association between Population Exposure to Extreme Temperature and Burden of Disease Attributed to Non-Optimal Temperatures

Older and child populations exposed to extreme hot and cold temperatures were both positively and significantly associated with death rate and YLL, after controlling for SES, medical resources, air quality, and climate characteristics (*p* < 0.01). The contemporary and cumulative impacts of extreme temperature are summarized in Table 2. The contemporary effects were calculated from base data estimating the effects of current year exposure on health in the same period. The cumulative effect refers to effects that will last into the future, which were calculated using the dynamic panel model. The effect sizes of the older population and children exposed to extreme heat were 2.703 (95% CI: 2.158–3.247) and 2.880 (95% CI: 2.330–3.430), respectively. Against the backdrop of global aging, if older population exposure to extreme heat were to maintain a temporal trend (0.52%) similar to that in 2010–2019, contemporary deaths attributed to high temperature would increase by 3904 per 10 million people in the next decade. The cumulative effects of population exposure to extreme heat (older population: 4.625 [95% CI: 4.584–4.667]; children: 5.618 [95% CI: 5.572–5.664]) were slightly larger than the contemporary effects. The contemporary effects were very similar to the cumulative effects, suggesting the effect of extreme heat rapidly decreased over time.

The effect sizes of the older population and children exposed to extreme cold were 6.576 (95% CI: 4.724–8.428) and 6.633 (95% CI: 4.783–8.482), respectively. Among the 20 countries, the older population exposed to extreme cold exhibited a rising trend. For these countries, assuming a similar upward temporal trend of older population exposure to that experienced in the last decade (0.67%), contemporary deaths attributed to low temperatures will increase by 185 per 10 million older people in the coming 10 years. Moreover, the cumulative effects of extreme cold increased (older population: 46.612 [95% CI: 46.352–46.874]; children: 48.257 [95% CI: 47.987–48.529]). Similar results were obtained when the effects on the YLL rate were estimated. As shown in Table S2, we further estimated the association between population exposure and mortality and YLL for CVD and CRD. As population exposure to extreme heat and cold also contributes to the burden of CVD and CRD, judging from the coefficient and 95% CI, which barely overlapped, the effect size on CVD was stronger than that of CRD.

In Table 3, we stratified our results by income group. In both HICs and LMICs, the greater the population exposure to extreme temperatures, the greater the health burden. Nevertheless, the health effects of population exposure to extreme events differ according to income level. The effects of population exposure to extreme heat on the burden of disease were significantly lower in HICs than in LMICs, whereas the health effects of population exposure to extreme cold were not significantly different from those in HICs. As a robustness check, mortality and YLL for CVD and CRD were divided by age group (e.g., age-standardized rate, children (under 5), and the elderly (age 55+)); the results were consistent (Tables S3 and S4).

## 4. Discussion

### 4.1. Association between Population Exposure to Extreme Temperatures and Disease Burden

In the context of global climate change, using worldwide data covering 171 countries, this study assessed vulnerable population exposure to extreme temperatures factoring out population growth and net change in demographic distributions; then, it provided the first estimation of the association between population exposure and disease burden. Consistent with the previous literature, which assessed the health effects of extreme temperature exposure, this study found that population exposure to extreme temperature contributed to rising mortality and YLL rates, with a stronger effect on CVD-attributed burden [5,20]. The potential biophysical mechanism suggests that extreme temperature places an added demand on body temperature regulation, with excess blood diverted underneath the skin, which could cause overloaded stress on the heart and lungs. Both extreme heat and cold could increase blood viscosity and plasma-cholesterol concentration, triggering heart failure, myocardial ischemia, and stroke. In contrast, breathing both cold dry and hot humid air can trigger bronchoconstriction, which could exacerbate CRD [6,15,26].

Furthermore, health risks stemming from extreme temperatures could accumulate to a greater extent. After the deduction of contemporary effects, the lag effect for extreme heat decreased rapidly, whereas the lag effects of extreme cold developed to a much greater extent. Several studies have documented a similar lagged response to extreme temperatures. For example, a study by Yang et al. [27] used data from Shanghai, China and reported that the effects of extremely low temperatures were delayed and persisted for 2 weeks, while extreme heat effects were limited to the first 5 days, followed by a significant mortality displacement (9 days). Another study by Pascal et al. [28] also illustrated a similar temporal dynamic of the temperature mortality response to high- and low-temperature exposure using data from 18 cities in France. Under the situation of increased cardiac workload, a series of acute cardiovascular responses to the failure of body thermoregulation could occur within a short duration of heat effects. For example, dehydration, salt depletion, and local and systemic acute inflammatory responses may trigger heart failure. Furthermore, a higher risk of thrombosis due to extreme heat may lead to myocardial ischemia and stroke. In contrast, in addition to an acute cardiac response, extreme cold may lead to myocardial ischemia or myocardial infarction in the future because of the prolonged impact of cold on the human cardiovascular system [29]. Extreme cold may activate the sympathetic nervous system and increase catecholamine secretion, in turn elevating heart rate, peripheral vascular resistance, and blood pressure. All these changes could indirectly decrease the ratio of myocardial oxygen supply to demand and eventually lead to myocardial ischemia or myocardial infarction [29].

### 4.2. Association between Population Exposure to Extreme Temperatures and Disease Burden between HICs and LMICs

Notably, population exposure and corresponding health risks varied between HICs and LMICs. Population exposure to extreme cold and total health loss attributed to extreme cold in HICs were greater than those in LMICs, but the marginal effects of population exposure to extreme cold on attributed health burden were greater in LMICs. The population in LMICs was more likely to suffer from associated health risks compared to those in HICs, once they were exposed to extreme cold. In contrast, population exposure to extreme heat was more likely to occur in LMICs in 2010–2019, and the attributed health loss was greater in LMICs. However, no significant differences were observed in the health effects of exposure to extreme heat between HICs and LMICs. For HICs, they may want to ultimately reduce the prevalence of extreme cold, while LMICs may need to decrease the prevalence of extreme heat, as well as reducing the marginal effects of extreme cold.

Several studies have found that extreme temperature-related mortality is largely preventable through behavioral adaptations [30,31], including the use of air conditioning [32,33], increased fluid intake [34], and improved healthcare [8,32,35,36]. Nevertheless, previous studies have found that SES moderates the association between extreme temperatures and health risks, and low-SES countries or people are the most affected by extreme temperature events [11,17,32,36]. Those with high SES can cope with extreme temperatures with sufficient equipment, human resources, and technologies [11,17,36]. Judging from the association between population exposure and health loss, the vulnerable population in HICs was less likely to suffer health risks from extreme cold [11,17]. However, this does not imply an absolutely disadvantaged status in LMICs. This study also documented that the effects of population exposure to extreme heat were similar between LMICs and HICs, even though LMICs presented a large number of exposed population. A potential explanation for this may be that they developed a set of effective adaptation behaviors after experiencing high frequencies of exposure [11,31]. Similarly, health loss from extreme cold could also be reduced by implementing effective interventions in LMICs [11].

### 4.3. Contribution and Limitations

This is the first study to assess the association between the exposure of vulnerable populations to extreme temperatures and disease burden, and will help to enhance a globally comprehensive understanding of health risks resulting from population exposure to extreme temperatures. Furthermore, the study compared the effects of population exposure between LMICs and HICs, helping to identify targeted strategies for dealing with extreme temperatures by country. This study has several limitations. First, as an empirical study, the study lacked a comprehensive understanding of the mechanisms linking population exposure to extreme temperatures with health risks. For example, we cannot determine whether the different effects of economic disparities are really caused by income, or are they caused by geographical differences in ambient temperatures. Low- and middle-income countries are often located in warmer climates, and thus also suffer from greater extremes of temperature compared to high-income countries. Geographic location, rather than economic factors, may contribute to differences in the effects of extreme temperature between low- and middle-income countries and high-income countries. Second, we used mortality and the YLL rate to measure health risks, which does not fully capture the health impacts of extreme temperatures, especially on morbidity and life expectancy. Third, the study focused on the health effects of extreme temperatures, leaving open the effects of other types of extreme events, including widespread flooding, extreme precipitation, long periods of drought and wet periods, and storms. It is important to understand the extent to which these events would affect health by interacting with temperature changes. Fourth, the GBD 2019 might have underestimated the global temperature-related mortality burden, which was gauged based on data from eight countries [9]. Fifth, previous studies have shown that populations with extreme ages are more prone to heat effects, while the health effects of extreme cold can be seen in all age groups [37]. The attributable disease burden due to extreme cold temperature for all age groups was not assessed in this study, which may have resulted in the underestimation of the effects of extreme cold exposure. Sixth, this study estimated the association between population exposure to extreme temperature and attributed disease burden using national data, and thus the study was not able to exactly match local mortality statistics to regional/local temperature values. Nevertheless, temperature varies geographically within countries and has different impacts on people depending on where they live. Although we try to reduce the issue using the weighted population exposure to extreme heat, local-level data might be better for this type of research question than national-level statistics. Unfortunately, such data are not available globally. We will try to collect local-level data and re-run the regression in the future. Seventh, our results may be biased due to sample selection, since 25 countries (Andorra, Greece, Liechtenstein, Monaco, Palau, Saint Kitts and Nevis, San Marino, United Kingdom of Great Britain and Northern Ireland, Comoros, Mali, Somalia, Kyrgyzstan, Ukraine, Uzbekistan, Albania, Belarus, Bulgaria, Dominica, Marshall Islands, Nauru, Turkmenistan, Tuvalu, Cook Islands, Holy See and Niue) did not have data available for their health systems for a period of more than five years between 2010 and 2019. Our sample excluded such countries. According to the 2017 World Bank classification by income level, these 25 countries include eight high-income countries, fourteen low- and middle-income countries, and three unclassified countries. According to their geographic location, there are fourteen European countries located in the temperate zone, while the others are tropical countries, distributed in Oceania (six countries), Africa (three countries) and North America (two countries). Although the excluded countries seem to be random, sample bias could be a concern in our regression results. More data should be collected to test the effects of population exposure to extreme temperatures.

## 5. Conclusions

Based on data from 171 countries in the last decade, the study investigated vulnerable population exposure to extreme temperature and its association with the burden of disease. Increased exposure of vulnerable populations to extreme temperature contributes to burden of disease, measured on the basis of mortality and YLL rate. Furthermore, the cumulative effects of extreme cold were greater than the contemporary effects. The population distribution of extreme temperature varied by country. Extreme cold affected more people in HICs, while extreme heat occurred more in LMICs. However, the association between population exposure to extreme cold and burden of disease was stronger in LMICs than in HICs, while the effect sizes of population exposure to extreme heat was similar for the two countries. Our study highlighted that reducing extreme temperature events and enhancing adaption capacity should be paid attention simultaneously, and LMICs should invest more in reducing the prevalence of extreme heat events and enhancing the capacity to adapt to extreme cold.

## Figures and Tables

**Table 1:** Descriptive statistics.

Variables	Total (*n* = 1710) Median (IQR ^b^)	HICs ^c^ (*n* = 510) Median (IQR)	LMICs ^d^ (*n* = 1200) Median (IQR)	*p*-Value ^e^
IHR ^a^ capacity scores (%)	69 (51, 85)	85 (73, 92)	61 (45, 76)	0.000
Urban population (% of total population)	57.08 (36.85, 76.18)	81.11 (67.11, 88.55)	48.43 (32.21, 64.14)	0.000
GDP per capita, PPP (current international 1000$)	11.73 (4.02, 26.77)	39.84 (28.91, 50.58)	6.81 (2.85, 12.71)	0.000
Ambient particulate matter pollution (ug/m^3^)	23.44 (15.76, 39.11)	14.64 (9.79, 20.04)	29.04 (20.73, 43.33)	0.000
Older population exposure to extreme heat (person-days/year^−1^)	128.30 (42.98, 248.30)	33.73 (9.92, 87.92)	179.80 (87.95, 275.30)	0.000
Older population exposure to extreme cold (person-days/year^−1^)	0.00 (0.00, 19.79)	20.36 (0.45, 72.81)	0.00 (0.00, 2.26)	0.000
Children exposure to extreme heat (person-days/year^−1^)	124 (42.06, 241.10)	32.47 (9.80, 90.09)	166.10 (85.17, 264.90)	0.000
Children exposure to extreme cold (person-days/year^−1^)	0.00 (0.00, 18.44)	18.39 (0.42, 71.57)	0.00 (0.00, 2.12)	0.000
Age-standardized mortality attributable to high temperature (per 10 million population)				
All causes	212.20 (19.17, 567.30)	9.43 (0.93, 240.50)	278.40 (98.81, 747.30)	0.000
Cardiovascular diseases	30.43 (2.61, 170.80)	4.03 (0.44, 33.76)	54.24 (10.73, 243)	0.000
Chronic respiratory diseases	0.46 (−0.10, 7.64)	0.02 (−0.02, 1.60)	0.96 (−0.36, 10.33)	0.001
Age-standardized mortality attributable to low temperature (per 10 million population)				
All causes	1443 (381.30, 2505)	1778 (1256, 2281)	1070 (313.20, 2766)	0.000
Cardiovascular diseases	948.80 (385.10, 1717)	1057 (773.20, 1493)	813 (320.70, 1846)	0.005
Chronic respiratory diseases	148.60 (43.62, 366.50)	275.80 (122.50, 431.10)	113.20 (38.73, 310.40)	0.000
Age-standardized YLL ^f^ attributable to high temperature (per 100,000 population)				
All causes	61.64 (4.54, 175.90)	1.98 (0.22, 60.17)	85.81 (31.02, 231.80)	0.000
Cardiovascular diseases	5.62 (0.42, 32.39)	0.62 (0.07, 5.64)	10.12 (2.02, 47.62)	0.000
Chronic respiratory diseases	0.08 (−0.02, 1.33)	0.00 (0.00, 0.24)	0.16 (−0.06, 1.79)	0.000
Age-standardized YLL attributable to low temperature (per 100,000 population)				
All causes	190.70 (46, 366.60)	202.90 (120.50, 289.70)	166.30 (39.10, 445.80)	0.627
Cardiovascular diseases	153.20 (71.92, 304)	156.20 (110.90, 226.80)	148.60 (60.43, 335)	0.619
Chronic respiratory diseases	24.54 (7.48, 59.85)	41.68 (18.16, 66.16)	19.82 (6.55, 50.85)	0.000

^a^ IHR: International Health Regulations. ^b^ IQR: interquartile range. ^c^ HICs: high-income countries. ^d^ LMICs: low-income and middle-income countries. ^e^ Applying the non-parametric Mann–Whitney U test. ^f^ YLL: years of life lost.

**Table 2:** Contemporary and cumulative association between population exposure to extreme temperature and all-cause health risks.

	Variables	Health Outcomes Attributable to High Temperature		Health Outcomes Attributable to Low Temperature	
		Mortality	YLL ^a^	Mortality	YLL
		Coef. (95%CI ^b^)	Coef. (95%CI)	Coef. (95%CI)	Coef. (95%CI)
Contemporary effects	Older population exposure to extreme heat	2.703 ***	0.683 ***		
		(2.158–3.247)	(0.515–0.851)		
	Children exposure to extreme heat	2.880 ***	0.725 ***		
		(2.330–3.430)	(0.569–0.881)		
	Older population exposure to extreme cold			6.576 ***	0.931 ***
				(4.724–8.428)	(0.566–1.296)
	Children exposure to extreme cold			6.633 ***	0.918 ***
				(4.783–8.482)	(0.562–1.275)
Cumulative effects	Lag of independent variable	0.936 ***	0.929 ***		
		(0.911–0.962)	(0.898–0.959)		
	Older population exposure to extreme heat	0.294 ***	0.069 **		
		(0.099–0.489)	(0.016–0.122)		
	Cumulative effects	4.625	0.965		
		(4.584–4.667)	(0.866–1.075)		
	Lag of independent variable	0.924 ***	0.913 ***		
		(0.894–0.953)	(0.879–0.948)		
	Children exposure to extreme heat	0.428 ***	0.108 ***		
		(0.174–0.682)	(0.039–0.178)		
	Cumulative effects	5.618	1.251		
		(5.572–5.664)	(1.139–1.374)		
	Lag of independent variable			0.987 ***	0.981 ***
				(0.981–0.993)	(0.976–0.986)
	Older population exposure to extreme cold			0.598 **	0.166 **
				(0.030–1.167)	(0.026–0.305)
	Cumulative effects			46.612	8.809
				(46.352–46.874)	(8.766–8.852)
	Lag of independent variable			0.987 ***	0.981 ***
				(0.981–0.993)	(0.976–0.986)
	Children exposure to extreme cold			0.615 **	0.170 **
				(0.041–1.189)	(0.022–0.317)
	Cumulative effects			48.257	9.029
				(47.987–48.529)	(8.985–9.073)

^a^ YLL: years of life lost. ^b^ CI: confidence interval. *** *p* < 0.01, ** *p* < 0.05.

**Table 3:** Association between population exposure to extreme temperature and mortality and YLL for HICs and LMICs.

Variables	Mortality Attributable to High Temperature						YLL ^e^ Attributable to High Temperature					
	All Causes		Cardiovascular Diseases		Chronic Respiratory Diseases		All Causes		Cardiovascular Diseases		Chronic Respiratory Diseases	
	HICs ^a^	LMICs ^b^	HICs	LMICs	HICs	LMICs	HICs	LMICs	HICs	LMICs	HICs	LMICs
	Coef. (95%CI ^c^)	Coef. (95%CI)	Coef. (95%CI)	Coef. (95%CI)	Coef. (95%CI)	Coef. (95%CI)	Coef. (95%CI)	Coef. (95%CI)	Coef. (95%CI)	Coef. (95%CI)	Coef. (95%CI)	Coef. (95%CI)
Older population exposure to extreme heat	2.686 ***	2.630 ***	0.908 ***	0.743 ***	0.031 ***	0.028 ***	0.733 ***	0.657 ***	0.182 ***	0.126 ***	0.005 ***	0.004 ***
	(1.663–3.710)	(2.045–3.215)	(0.448–1.367)	(0.557–0.928)	(0.014–0.049)	(0.009–0.047)	(0.433–1.033)	(0.470–0.844)	(0.077–0.286)	(0.068–0.184)	(0.003–0.008)	(0.001–0.007)
Empirical *p*-values ^d^	0.473		0.249		0.451		0.336		0.147		0.314	
Children exposure to extreme heat	2.727 ***	2.825 ***	0.924 ***	0.821 ***	0.032 ***	0.029 ***	0.736 ***	0.707 ***	0.182 ***	0.142 ***	0.005 ***	0.004 ***
	(1.636–3.817)	(2.232–3.418)	(0.441–1.407)	(0.634–1.007)	(0.015–0.049)	(0.008–0.049)	(0.418–1.053)	(0.537–0.877)	(0.074–0.290)	(0.088–0.196)	(0.003–0.008)	(0.001–0.007)
Empirical *p*-values	0.438		0.343		0.454		0.428		0.226		0.320	
	**Mortality attributable to low temperature**						**YLL attributable to low temperature**					
Older population exposure to extreme cold	4.939 ***	8.890 ***	4.327 ***	6.063 ***	0.801 ***	2.201 **	0.481 ***	1.712 ***	0.662 ***	1.083 ***	0.122 ***	0.347 **
	(3.641–6.238)	(3.642–14.138)	(3.230–5.424)	(2.663–9.463)	(0.450–1.152)	(0.471–3.931)	(0.234–0.727)	(0.624–2.800)	(0.439–0.886)	(0.492–1.675)	(0.060–0.185)	(0.074–0.620)
Empirical *p*-values	0.021		0.107		0.013		0.000		0.045		0.008	
Children exposure to extreme cold	4.969 ***	9.062 ***	4.353 ***	6.453 ***	0.810 ***	2.113 **	0.479 ***	1.704 ***	0.663 ***	1.146 ***	0.123 ***	0.334 **
	(3.651–6.287)	(3.739–14.385)	(3.233–5.473)	(2.875–10.031)	(0.448–1.172)	(0.416–3.810)	(0.232–0.726)	(0.620–2.788)	(0.436–0.890)	(0.522–1.770)	(0.059–0.188)	(0.066–0.602)
Empirical *p*-values	0.016		0.069		0.018		0.000		0.028		0.015	

^a^ HICs: high-income countries. ^b^ LMICs: low-income and middle-income countries. ^c^ CI: confidence interval. ^d^ High-income countries group versus low-income and middle-income countries group. ^e^ YLL: years of life lost. *** *p* < 0.01, ** *p* < 0.05.

## Data Availability

The climate-related data can be obtained from ERA5-HEAT https://cds.climate.copernicus.eu/cdsapp#!/dataset/10.24381/cds.553b7518?tab=overview (accessed on 25 December 2021), and the population data can be obtained from Socioeconomic Data and Applications Center Gridded Population of the World version 4 https://beta.sedac.ciesin.columbia.edu/data/collection/gpw-v4 (accessed on 15 November 2021) and the United Nations World Population Prospects https://population.un.org/wpp/Download/Standard/Population/ (accessed on 2 November 2021), whereas mortality and YLL related can be obtained from the Global Burden of Disease Study (GBD 2019) Results Tool database http://ghdx.healthdata.org/gbd-results-tool (accessed on 8 March 2022).

## Supplementary Materials

The following supporting information can be downloaded at: https://www.mdpi.com/article/10.3390/ijerph192013288/s1. Text S1: Calculation of disease burden attributed to non-optimal temperature; Text S2: Calculation of population exposure to extreme heat and cold; Text S3: Measurements of SES and air pollution exposure; Text S4: Multiple imputation; Table S1: Descriptive statistics; Table S2: Association between population exposure to extreme temperature and CVD/CRD mortality and YLL; Table S3: Association between older population exposure to extreme temperature and health risks for the elderly; Table S4: Association between children exposure to extreme temperature and health risks for the children. References [3,24,38,39,40,41] are cited in the Supplementary Materials.

## Abbreviations

HICs: High-income countries; LMICs: Low-income and middle-income countries; YLL: Years of life lost; GBD: Global Burden of Disease Study; SES: Socioeconomic status; IHR: International Health Regulations; UTCI: Universal climate thermal index; GMM: Generalized Method of Moments; CI: Confidence intervals; CVD: Cardiovascular diseases; CRD: Chronic respiratory diseases; IQR: Interquartile range.
