# Supplementary material for: Global Population Exposure to Extreme Temperatures and Disease Burden
Source: Int J Environ Res Public Health. 2022 Oct 14;19(20):13288. doi: 10.3390/ijerph192013288 (PMC9603138; doi:10.3390/ijerph192013288)
Supplement: Supplementary file 1 [file ijerph-19-13288-s001.zip › ijerph-1956929-supplementary.pdf]

International Journal of
Environmental Research and Public Health

MDPI

Article

Global Population Exposure to Extreme Temperatures and Disease Burden

Yajie Du [1,2,†], Ming Jing [3,†], Chunyu Lu [1,2], Jingru Zong [1,2], Lingli Wang [1,2] and Qing Wang [1,2,*]

[1] Department of Biostatistics, School of Public Health, Cheeloo College of Medicine, Shandong University, Jinan 250012, China
[2] National Institute of Health Data Science of China, Shandong University, Jinan 250012, China
[3] School of Computer Science and Technology, Qilu University of Technology (Shandong Academy of Science), Jinan 250353, China
* Correspondence: 201999000066@sdu.edu.cn
† These authors contributed equally to this work.

Supplementary Materials

Table of Contents

Text S1. Calculation of disease burden attributed to non-optimal temperature
Text S2. Calculation of population exposure to extreme heat and cold
Text S3. Measurements of SES and air pollution exposure
Text S4. Multiple imputation
Table S1. Descriptive statistics.
Table S2. Association between population exposure to extreme temperature and CVD/CRD mortality and YLL.
Table S3. Association between older population exposure to extreme temperature and health risks for the elderly.
Table S4. Association between children exposure to extreme temperature and health risks for the children.

Text S1. Calculation of Disease Burden Attributed to Non-Optimal Temperature

The Global Burden of Disease Study (GBD 2019) is well-recognized for the effort to examine attributable burden using standardized methods for a wide set of risk factors across all countries. In GBD 2019, measures of high and low non-optimal temperatures exposure were calculated for the first time. The all-cause diseases attributable to non-optimal temperature provided by the GBD 2019 was included twelve causes, that is ischaemic heart disease, stroke, hypertensive heart disease, diabetes, chronic kidney disease, lower respiratory infection, chronic obstructive pulmonary disease, homicide, suicide, mechanical injuries, transport-related injuries, and drowning. Cardiovascular diseases attributed to temperature extremes included ischaemic heart disease and hypertensive heart disease. Chronic respiratory diseases attributed to temperature extremes included lower respiratory infection and chronic obstructive pulmonary disease.

The definition of non-optimal temperature provided by the GBD 2019 is as follows: the same day exposure to ambient temperature that is either warmer or colder than the temperature associated with the minimum mortality risk. Specifically, the theoretical minimum risk exposure level for temperature is identified as the temperature that is associated with the lowest overall mortality attributable to the risk, in a given location and year.

Based on average daily temperature and temperature zone (defined by mean annual temperature), a robust meta-regression framework, implemented through the MR-BRT (meta-regression—Bayesian, regularized, trimmed) tool, was used to estimate cause-specific mortality. In each age-sex-location-year included, the theoretical mini-

mum risk exposure level for temperature was defined as the temperature associated with the lowest mortality for all included causes. Finally, attributable mortality and years of life lost (YLL) were computed by multiplying population attributable fractions by the relevant outcome quantity for each age-sex-location-year. The detailed methods to estimate the disease burden have been introduced in GBD 2019 report [38,39].

Text S2. Calculation of Population Exposure to Extreme Heat and Cold

ERA5-HEAT is computed using the ERA5 climate variables from the European Centre for Medium-Range Forecasts, which provides hourly universal thermal climate index (UTCI) records on regular latitude-longitude grids at 0.25° × 0.25° resolution [24]. The daily maximum and minimum UTCIs were calculated from the hourly data, and the numbers of days per year with extreme heat and extreme cold were added from the daily UTCI per grid cell.

To obtain the demographic distribution of vulnerable populations between 2010–2019, the spatial distribution of Socioeconomic Data and Applications Center Gridded Population of the World version 4 (GPWv4) were merged with the temporal trends of the United Nations World Population Prospects (UN WPP) [3]. The GPWv4 provides gridded demographics at 0.25° × 0.25° grid resolution for 2010, including total population and each 5-year age group population. The UN WPP provides country-level demographic data. From 2010 to 2019, the grid population data was adjusted annually to match the average population distribution of the corresponding grid cells for each country with country-level values. This allows the high spatial resolution data for 2010–2019 to be maintained on the simplified assumption that the relative spatial distribution of the population in each age group within each country remains approximately unchanged [40]. This was achieved as follows:

Step 1: For each country, the proportional change in fraction of demographic in each 5-year age group versus 2010 was computed as:

$$\delta^{wpp}_{year,country,age} = f^{wpp}_{year,country,age} / f^{wpp}_{2010,country,age} \quad \text{(S1)}$$

where: $\delta^{wpp}_{year,country,age}$ is the ratio of change in demographic for a given age group and country from the UN WPP dataset; $f^{wpp}_{year,country,age}$ is the fraction of population in the UN WPP dataset for a given age group, country and year; $f^{wpp}_{2010,country,age}$ is the fraction of population in the UN WPP dataset for a given age group and country for the year 2010.

Step 2: For each grid cell corresponding to a given country, the fraction of population in a given age group was calculated as:

$$f^{gpw}_{year,c,age} = \delta^{wpp}_{year,country,age} * f^{gpw}_{2010,c,age} \quad \text{(S2)}$$

where: $f^{gpw}_{year,c,age}$ is the fraction of the population in a given age group for a given year, for the grid cell c; $f^{gpw}_{2010,c,age}$ is the fraction of the population in a given age group for 2010, for the grid cell c.

Step 3: For the grid cell c, the population for a given age group and year $P^{gpw}_{year,c,age}$ was calculated as:

$$P^{gpw}_{year,c,age} = P^{gpw}_{year,c,total} * f^{gpw}_{year,c,age} \quad \text{(S3)}$$

$$P^{gpw}_{year,c,total} = P^{gpw}_{2010,c,total} * \delta^{wpp}_{year,country,total} \quad \text{(S4)}$$

$$\delta^{wpp}_{year,country,total} = P^{wpp}_{year,country,total} / P^{wpp}_{2010,country,total} \quad \text{(S5)}$$

where: $P^{gpw}_{year,c,age}$ is the population for a given age group and year, for the grid cell c; $P^{gpw}_{year,c,total}$ is the total population for a given year, for the grid cell c; $P^{gpw}_{2010,c,total}$ is the total population for 2010, for the grid cell c; $\delta^{wpp}_{year,country,total}$ is the ratio of change in total population for a given country and year versus 2010 from the UN WPP dataset; $P^{wpp}_{year,country,total}$ is the total population for a given country and year; $P^{wpp}_{2010,country,total}$ is the total population for a given country of year 2010.

Step 4: For each country, grid cells were matched with country code using the gridded country code lookup data and country name lookup table provided by the GPWv4 dataset.

Therefore, the exposure-weighted mean to extreme heat and cold for 171 countries from 2010 to 2019 could be calculated as follows:

$$exposure\ weighted\ mean^{country}_{year,age,heat} = \sum_c (D^{country}_{year,c,heat} * P^{gpw}_{year,c,age}) / P^{wpp}_{year,country,age} \quad (S6)$$

$$exposure\ weighted\ mean^{country}_{year,age,cold} = \sum_c (D^{country}_{year,c,cold} * P^{gpw}_{year,c,age}) / P^{wpp}_{year,country,age} \quad (S7)$$

where: $exposure\ weighted\ mean^{country}_{year,age,heat}$ is the exposure-weighted mean to extreme heat for a given age group, country and year; $D^{country}_{year,c,heat}$ is the number of extreme heat days for a given country and year, for the grid cell c; $P^{wpp}_{year,country,age}$ is the total population for a given age group, country and year. $exposure\ weighted\ mean^{country}_{year,age,cold}$ is the exposure-weighted mean to extreme cold for a given age group, country and year; $D^{country}_{year,c,cold}$ is the number of extreme cold days for a given country and year, for the grid cell c.

Text S3. Measurements of SES and Air Pollution Exposure

Measurements of socioeconomic status (SES) and air pollution exposure, such as urbanization (proportion of urban population), gross domestic product (GDP) per capita, the International Health Regulations (IHR) core capacities score, and particulate matter (PM) exposure, were included as determinants of health. Urbanization and GDP per capita for 217 economies from 2010 to 2019 were obtained from the World Development Indicators database of the World Bank (https://databank.worldbank.org/source/world-development-indicators). The data set provides relevant, high-quality, and internationally comparable statistics about global development.

The yearly ambient PM pollution data for 204 countries from 2010 to 2019 was acquired through GBD 2019 Air Pollution Exposure Estimates 1990–2019 (https://ghdx.healthdata.org/record/global-burden-disease-study-2019-gbd-2019-air-pollution-exposure-estimates-1990-2019). Exposure to ambient PM pollution was measured by the population-weighted annual average mass concentration of particles with an aerodynamic diameter less than 2.5 micrometers ($PM_{2.5}$) in a cubic meter of air. The Data Integration Model for Air Quality was used to estimate the indicator, with data coming from multiple sources, including satellite observations of aerosols in the atmosphere, ground measurements, chemical transport model simulations, population estimates, and land-use data. The detailed methods have been introduced in GBD 2019 report [38,39].

In addition, the IHR core capacities score was proposed by the World Health Organization (WHO) to reflect a country's potential capacity that detection, preparedness and response to public health risks and emergencies of national and international concern. The IHR scores data for 171 countries from 2010 to 2019 was collected from the

WHO IHR State Parties Self-Assessment Annual Reporting Tool (SPAR) (https://extranet.who.int/e-spar#capacity-score).

The IHR required that all countries have the ability to detect, assess, report, and respond to public health events. The status of IHR implementation from 2010 to 2017 was assessed through the IHR monitoring questionnaire. Since 2018, it has been submitted online by IHR SPAR, and some contents have been revised. For example, capacity C1.2 and C1.3 have been added to capacity C1 (National legislation and financing); The Response and Preparedness capabilities were integrated into a new capacity C8 (National health emergency framework); And a new capacity was added as C9 (Health service provision). The details are presented in the following tables:

(1) 2010–2017 version (IHR monitoring questionnaire).

Capacity	Capacity Title	Indicator	Indicator Title
C.1	National legislation, policy & financing	C.1.1.1	Laws, regulations, administrative requirements, policies or other government instruments in place, sufficient for implementation of obligations under the IHR
C.2	Coordination and NFP Communications	C.2.1.1	Mechanism established for the coordination of relevant sectors in the implementation of IHR
C.2	Coordination and NFP Communications	C.2.1.2	IHR NFP functions and operations in place as defined by IHR (2005)
C.3	Surveillance	C.3.1.1	Indicator-based (Routine) surveillance (IBS) includes early warning function for early detection of public health events
C.3	Surveillance	C.3.2.1	Event-Based Surveillance established
C.4	Response	C.4.1.1	Public Health Emergency Response mechanisms established
C.4	Response	C.4.2.1	Infection Control and Prevention (IPC) established at national and hospital levels
C.5	Preparedness	C.5.1.1	Multi-hazard National Public Health Emergency Preparedness and Response Plan exists
C.5	Preparedness	C.5.2.1	Public health risks and resources mapped
C.6	Risk Communication	C.6.1.1	Mechanisms for effective risk communication during a public health emergency are established
C.7	Human Resource Capacity	C.7.1.1	Human resources available to implement IHR Core Capacity requirements
C.8	Laboratory	C.8.1.1	Laboratory services available and accessible to test for priority health threats
C.8	Laboratory	C.8.2.1	Laboratory biosafety and biosecurity practices in place
C.9	Points of Entry	C.9.1.1	General obligations at PoE are fulfilled
C.9	Points of Entry	C.9.2.1	Effective surveillance established at PoE
C.9	Points of Entry	C.9.3.1	Effective response at PoE established
C.10	Zoonotic Events	C.10.1.1	Mechanisms for detecting and responding to zoonoses and potential zoonoses established
C.11	Food Safety	C.11.1.1	Mechanisms established for detecting and responding to foodborne disease and food contamination
C.12	Chemical Events	C.12.1.1	Mechanisms established for detection, alert and response to chemical emergencies
C.13	Radiation Emergencies	C.13.1.1	Mechanisms established for detecting and responding to radiological and nuclear emergencies

(2) 2018–2019 version (IHR SPAR).

Capacity	Capacity Title	Indicator	Indicator Title
C.1	Legislation and Financing	C.1.1	Legislation, laws, regulations, policy, administrative requirements or other government instruments to implement the IHR

C.1	Legislation and Financing	C.1.2	Financing for the implementation of IHR capacities
C.1	Legislation and Financing	C.1.3	Financing mechanism and funds for timely response to public health emergencies
C.2	IHR Coordination and National IHR Focal Point Functions	C.2.1	National IHR Focal Point functions under IHR
C.2	IHR Coordination and National IHR Focal Point Functions	C.2.2	Multisectoral IHR coordination mechanisms
C.3	Zoonotic Events and the Human–animal Interface	C.3.1	Collaborative effort on activities to address zoonoses
C.4	Food Safety	C.4.1	Multisectoral collaboration mechanism for food safety events
C.5	Laboratory	C.5.1	Specimen referral and transport system
C.5	Laboratory	C.5.2	Implementation of a laboratory biosafety and biosecurity regime
C.5	Laboratory	C.5.3	Access to laboratory testing capacity for priority diseases
C.6	Surveillance	C.6.1	Early warning function: indicator-and event-based surveillance
C.6	Surveillance	C.6.2	Mechanism for event management (verification, risk assessment, analysis investigation)
C.7	Human Resources	C.7.1	Human resources for the implementation of IHR capacities
C.8	National Health Emergency Framework	C.8.1	Planning for emergency preparedness and response mechanism
C.8	National Health Emergency Framework	C.8.2	Management of health emergency response operations
C.8	National Health Emergency Framework	C.8.3	Emergency resource mobilization
C.9	Health Service Provision	C.9.1	Case management capacity for IHR relevant hazards
C.9	Health Service Provision	C.9.2	Capacity for infection prevention and control and chemical and radiation decontamination
C.9	Health Service Provision	C.9.3	Access to essential health services
C.10	Risk communication	C.10.1	Capacity for emergency risk communications
C.11	Points of Entry	C.11.1	Core capacity requirements at all times for designated airports, ports and ground crossings
C.11	Points of Entry	C.11.2	Effective public health response at points of entry
C.12	Chemical Events	C.12.1	Resources for detection and alert
C.13	Radiation Emergencies	C.13.1	Capacity and resources

(3) Summary of the changes between two versions.

**Change**	**2010–2017 Version**	**2018–2019 Version**
Name change	IHR monitoring questionnaire	IHR State Party self-assessment Annual Reporting Tool
Name change	Core capacity	Capacity
Addition of indicators	National legislation, policy and financing	C1. National legislation and financing →Addition of financing indicators (C1.2 and C1.3)
Name change	Zoonotic events	C3. Zoonotic events and the human–animal interface
Merging of capacities	Response<br>Preparedness	C8. National health emergency framework
Addition of new indicators		C9. Health service provision
Structure	Yes/no/not known responses	Five progressive levels of capacity
Structure		Colour coded

Therefore, in order to maintain consistency of capacities for the period 2010–2019, we consolidated Response and Preparedness capacities for 2010–2017 into the National health emergency framework capacity, while the capacity C1.2, C1.3 and C9 (Health ser-

vice provision) for 2018 and 2019 were removed. Finally, we calculated the mean value of these 12 core capacities to represent the overall level of national health emergency response.

Text S4 Multiple Imputation

Multiple imputation (MI) with chained equations was used to impute any missing data by creating 10 imputed datasets. The missing values occurred mostly for control variables, including urbanization, gross domestic product per capita, and the International Health Regulations (IHR) core capacities score; there were no data missing for particulate matter exposure, exposure weighted mean of vulnerable population to extreme temperature and disease burden attributed to non-optimal temperature. Differences between individuals with complete and incomplete data are presented in Table 1a of the Text S4. Those with missing values were residents living in less developed countries. Missing health measurements were worse than measured health but only because low-income countries may be more likely to have missing health indicators. We thus assumed the missing data are Missing At Random. Hence, MI with chained equations was applied to impute any missing data. MI was performed using the R multivariate imputation by chained equation package [41].

The MI process was conditional on particulate matter exposure, exposure weighted mean of vulnerable population to extreme temperature and disease burden attributed to non-optimal temperature. All the missing data were continuous variables and were dealt with linear regression. Complete data were transformed back to their original scales before analysis. Ten imputed datasets were created. Density plots were provided for visual representations of the extent to which imputed values differ from observed values. According to the distribution of complete and imputed data, the set of imputed data closest to the complete data distribution was selected.

Table: descriptive statistics by incomplete and complete data:

Variables	Incomplete Data N = 368 Median (IQR [b])	Complete Data N = 1342 Median (IQR)	*p*-Value [c]
IHR[a] capacity scores (%)	65.08 (49.25, 83.67)	71.15 (52.83, 85.83)	0.443
Urban population (% of total population)	54.25 (34.66, 70.00)	57.83 (39.43, 77.38)	0.001
GDP per capita, PPP (current international 1000$)	8.5 (2.80, 17.26)	12.85 (4.45, 29.04)	0.000
Ambient particulate matter pollution (ug/m^3)	27.13 (18.06, 42.82)	22.75 (15.50, 38.04)	0.002
Older population exposure to extreme heat (person-days/year^{-1})	157.8 (53.15, 252.6)	122.30 (41.16, 244.70)	0.004
Older population exposure to extreme cold (person-days/year^{-1})	0.00 (0.00, 7.45)	0.03 (0.00, 24.25)	0.000
Children exposure to extreme heat (person-days/year^{-1})	146 (54.49, 251.50)	117.30 (39.75, 237.30)	0.002
Children exposure to extreme cold (person-days/year^{-1})	0.00 (0.00, 6.67)	0.02 (0.00, 23.32)	0.000
**Age-standardized mortality attributable to high temperature (per 10 million population)**			
All causes	254 (33.51, 799.20)	191.50 (16.35, 534.50)	0.001
Cardiovascular diseases	39.55 (5.39, 265.30)	27.39 (1.80, 155.40)	0.010
Chronic respiratory diseases	1.21 (−0.01, 9.27)	0.30 (−0.11, 7.26)	0.002
**Age-standardized mortality attributable to low temperature (per 10 million population)**			
All causes	1146 (336.30, 2429)	1509 (413.20, 2565)	0.192
Cardiovascular diseases	854.60 (349.50, 1676)	962.60 (404.30, 1737)	0.438
Chronic respiratory diseases	113.30 (39.64, 314.80)	162.70 (45.99, 376.30)	0.011
**Age-standardized YLL [d] attributable to high temperature (per 100,000 population)**			
All causes	75.83 (9.31, 252.70)	58.87 (3.58, 165.10)	0.001
Cardiovascular diseases	7.09 (1.04, 50.63)	5.04 (0.33, 29.96)	0.008
Chronic respiratory diseases	0.20 (0.00, 1.57)	0.05 (−0.02, 1.20)	0.001

**Age-standardized YLL attributable to low temperature (per 100,000 population)**			
All causes	174.30 (35.82, 370)	193.80 (53.28, 366.60)	0.406
Cardiovascular diseases	150.20 (65.60, 294.60)	153.80 (74.30, 305.70)	0.740
Chronic respiratory diseases	19.40 (6.72, 52.90)	26.19 (7.59, 60.84)	0.027

[a.] IHR: International Health Regulations. [b.] IQR: interquartile range. [c.] Applying the non-parametric Mann-Whitney U test. [d.] YLL: years of life lost.

Table S1. Descriptive statistics.

Variables	Total (N = 1710) Median (IQR [a])	HICs [b] (N = 510) Median (IQR)	LMICs [c] (N = 1200) Median (IQR)	*p*-Value [d]
**Death rate attributable to high temperature in population ages over 55 (per 10 million population)**				
All causes	600.40 (59.90, 1918)	42.54 (4.03, 821.20)	855.60 (258.60, 2313)	0.000
Cardiovascular diseases	133.30 (11.85, 735.40)	21.61 (2.57, 144.30)	230.50 (43.60, 996.80)	0.000
Chronic respiratory diseases	2.17 (−0.49, 32.56)	0.10 (−0.15, 8.99)	4.20 (−1.36, 44.13)	0.000
**Death rate attributable to low temperature in population ages over 55 (per 10 million population)**				
All causes	5866 (1809, 12,622)	9655 (5621, 14,487)	4543 (1517, 10,061)	0.000
Cardiovascular diseases	3879 (1549, 7364)	5664 (3202, 7561)	3102 (1314, 7082)	0.000
Chronic respiratory diseases	609.60 (185.60, 1698)	1432 (409.20, 2599)	489.20 (167.20, 1113)	0.000
**Death rate attributable to high temperature in children under 5 (per 10 million population)**				
All causes	71.15 (2.93, 258.80)	0.57 (0.04, 34.78)	127.80 (40.98, 454.90)	0.000
Cardiovascular diseases	0.33 (0.00, 1.44)	0.00 (0.00, 0.17)	0.61 (0.13, 2.74)	0.000
Chronic respiratory diseases	0.00 (0.00, 0.04)	0.00 (0.00, 0.00)	0.01 (0.00, 0.06)	0.000
**Death rate attributable to low temperature in children under 5 (per 10 million population)**				
All causes	0.55 (−20.66, 119.90)	−2.88 (−18.64, 10.28)	8.41 (−23.21, 245.50)	0.000
Cardiovascular diseases	1.91 (0.69, 4.69)	1.26 (0.39, 2.15)	2.62 (0.83, 6.04)	0.000
Chronic respiratory diseases	0.50 (0.19, 1.52)	0.39 (0.21, 0.87)	0.63 (0.18, 1.84)	0.000
**YLL[e] rate attributable to high temperature in population ages over 55 (per 100,000 population)**				
All causes	114.80 (10.37, 347.30)	5.86 (0.57, 137.90)	161.10 (49.57, 438)	0.000
Cardiovascular diseases	22.88 (1.64, 126.20)	2.85 (0.33, 22.37)	41.80 (7.95, 182.90)	0.000
Chronic respiratory diseases	0.36 (−0.07, 5.29)	0.02 (−0.02, 1.27)	0.74 (−0.22, 7.45)	0.000
**YLL rate attributable to low temperature in population ages over 55 (per 100,000 population)**				
All causes	958.50 (327.90, 1793)	1315 (708.90, 1807)	814.20 (267.10, 1784)	0.000
Cardiovascular diseases	659 (277.50, 1194)	756.80 (415.20, 1036)	554.40 (230.90, 1283)	0.061
Chronic respiratory diseases	108.10 (31.15, 263.50)	201.60 (66.35, 351.60)	84.26 (28.37, 186.70)	0.000
**YLL rate attributable to high temperature in children under 5 (per 100,000 population)**				
All causes	62.29 (2.55, 226.20)	0.49 (0.04, 30.48)	111.90 (35.86, 399.40)	0.000
Cardiovascular diseases	0.29 (0.00, 1.27)	0.00 (0.00, 0.15)	0.54 (0.12, 2.38)	0.000
Chronic respiratory diseases	0.00 (0.00, 0.03)	0.00 (0.00, 0.00)	0.00 (0.00, 0.05)	0.000
**YLL rate attributable to low temperature in children under 5 (per 100,000 population)**				
All causes	0.99 (−17.52, 107.70)	−2.35 (−15.71, 9.46)	8.35 (−19.76, 218.10)	0.000
Cardiovascular diseases	1.67 (0.61, 4.08)	1.09 (0.34, 1.89)	2.29 (0.73, 5.28)	0.000
Chronic respiratory diseases	0.43 (0.17, 1.31)	0.34 (0.18, 0.75)	0.54 (0.16, 1.59)	0.000

[a.] IQR: interquartile range. [b.] HICs: high-income countries. [c.] LMICs: low-income and middle-income countries. [d.] Applying the non-parametric Mann-Whitney U test. [e.] YLL: years of life lost.

Table S2. Association between population exposure to extreme temperature and CVD/CRD mortality and YLL.

Variables	Health Outcomes Attributable to High Temperature			
	Mortality		YLL [d]	
	CVD [a] Coef. (95%CI [c])	CRD[b] Coef. (95%CI)	CVD Coef. (95%CI)	CRD Coef. (95%CI)
Older population exposure to extreme heat	0.798 *** (0.611–0.985)	0.030 *** (0.014–0.047)	0.140 *** (0.088–0.192)	0.004 *** (0.002–0.007)
Children exposure to extreme heat	0.870 *** (0.676–1.064)	0.031 *** (0.013–0.048)	0.154 *** (0.104–0.204)	0.004 *** (0.002–0.007)
	**Health outcomes attributable to low temperature**			
Older population exposure to extreme cold	5.205 *** (3.840–6.570)	1.271 *** (0.669–1.873)	0.895 *** (0.648–1.142)	0.206 *** (0.111–0.302)
Children exposure to extreme cold	5.351 *** (3.961–6.741)	1.236 *** (0.651–1.820)	0.917 *** (0.666–1.168)	0.201 *** (0.109–0.293)

[a.] CVD: cardiovascular diseases. [b.] CRD: chronic respiratory diseases. [c.] CI: confidence intervals. [d.] YLL: years of life lost. *** $p < 0.01$.

Table S3. Association between older population exposure to extreme temperature and health risks for the e[illegible]

Variables	All causes			Cardiovascular Diseases			[illegible]
	**Total** Coef. (95%CI [c])	**HICs** [a] Coef. (95%CI)	**LMICs** [b] Coef. (95%CI)	**Total** Coef. (95%CI)	**HICs** Coef. (95%CI)	**LMICs** Coef. (95%CI)	[illegible]
	**Mortality Attributable to High Temperature in Population Ages over 5[illegible]**						
Older population exposure to extreme heat	9.137 *** (7.100–11.173)	11.295 *** (6.454–16.137)	8.485 *** (6.456–10.514)	3.335 *** (2.451–4.218)	5.065 *** (2.151–7.979)	2.923 *** (2.191–3.654)	[illegible]
Empirical *p*-values [d]	0.147			0.042			
	**Mortality attributable to low temperature in population ages over 55**						
Older population exposure to extreme cold	32.921 *** (23.807–42.036)	25.780 *** (19.072–32.487)	44.149 *** (18.154–70.145)	24.714 *** (17.720–31.708)	19.456 *** (14.815–24.096)	32.388 *** (12.088–52.688)	[illegible]
Empirical *p*-values	0.025			0.030			
	**YLL [e] attributable to high temperature in population ages over 55**						
Older population exposure to extreme heat	1.576 *** (1.096–2.056)	2.029 *** (1.089–2.968)	1.435 *** (0.918–1.951)	0.535 *** (0.345–0.724)	0.869 *** (0.327–1.412)	0.452 *** (0.266–0.638)	[illegible]
Empirical *p*-values	0.123			0.027			
	**YLL attributable to low temperature in population ages over 55**						
Older population exposure to extreme cold	5.434 *** (3.874–6.994)	3.326 *** (2.133–4.519)	7.892 *** (3.456–12.328)	4.151 *** (2.962–5.339)	2.657 *** (1.687–3.627)	5.874 *** (2.611–9.136)	[illegible]
Empirical *p*-values	0.003			0.002			

[a] HICs: high-income countries. [b] LMICs: low-income and middle-income countries. [c] CI: confidence interval[illegible] low-income and middle-income countries group. [e] YLL: years of life lost. *** $p < 0.01$.

Table S4. Association between children exposure to extreme temperature and health risks for the children.

Variables	All Causes			Cardiovascular Diseases			[illegible]
	**Total** Coef. (95%CI [c])	**HICs** [a] Coef. (95%CI)	**LMICs** [b] Coef. (95%CI)	**Total** Coef. (95%CI)	**HICs** Coef. (95%CI)	**LMICs** Coef. (95%CI)	**Total** [illegible]
	**Mortality Attributable to High Temperature in Children under 5**						
Children exposure to extreme heat	1.549 *** (0.874–2.225)	0.294 *** (0.121–0.467)	1.831 *** (0.921–2.741)	0.011 *** (0.007–0.016)	0.006** (0.001–0.012)	0.013 *** (0.007–0.019)	[illegible]
Empirical *p*-values [d]	0.014			0.110			
	**Mortality attributable to low temperature in children under 5**						

Children exposure to extreme cold	1.126 * (−0.053–2.304)	−0.010 (−0.108–0.088)	5.049 ** (1.221–8.878)	0.003 (−0.011–0.017)	0.004 ** (0.001–0.008)	0.016 (−0.023–0.055)	0.001 (−0.001–0.003)	0.002 *** (0.001–0.003)	0.002 (−0.003–0.006)
Empirical *p*-values	0.000			0.233			0.396		
	**YLL [e] attributable to high temperature in children under 5**								
Children exposure to extreme heat	1.039 *** (0.585–1.493)	0.244 *** (0.098–0.390)	1.210 *** (0.631–1.789)	0.008 *** (0.004–0.011)	0.006 ** (0.001–0.010)	0.009 *** (0.005–0.013)	0.000 ** (0.000–0.000)	0.000 *** (0.000–0.000)	0.000 (−0.000–0.000)
Empirical *p*-values	0.028			0.231			0.401		
	**YLL attributable to low temperature in children under 5**								
Children exposure to extreme cold	0.931 * (−0.132–1.994)	−0.035 (−0.138–0.067)	4.873 *** (1.265–8.482)	0.002 (−0.011–0.014)	0.003 * (−0.001–0.006)	0.013 (−0.022–0.049)	0.000 (−0.002–0.002)	0.001 ** (0.000–0.003)	0.001 (−0.003–0.004)
Empirical *p*-values	0.000			0.224			0.415		

[a.] HICs: high-income countries. [b.] LMICs: low-income and middle-income countries. [c.] CI: confidence intervals. [d.] high-income countries group versus low-income and middle-income countries group. [e.] YLL: years of life lost. *** $p < 0.01$, ** $p < 0.05$, * $p < 0.1$.
